# Traumatic childhood events of parents enrolled in the Avon Longitudinal Study of Parents and Children (ALSPAC)

**DOI:** 10.12688/wellcomeopenres.15804.1

**Published:** 2020-04-08

**Authors:** Genette Ellis, Yasmin Iles-Caven, Kate Northstone, Jean Golding

**Affiliations:** 1 Centre for Academic Child Health, Bristol Medical School, University of Bristol, Bristol, BS8 2BN, UK; 2 Population Health Sciences, Bristol Medical School, University of Bristol, Bristol, BS8 2BN, UK

**Keywords:** ALSPAC, trauma, parent, child, childhood, behaviour, stress, longitudinal cohort

## Abstract

**Background**: Early life experiences can have a significant impact on an individual’s later behaviour, the way they view the world, their beliefs and their success at forming strong interpersonal relationships. These factors may subsequently influence the way that the individual may parent their children, which in turn may have an effect on their child’s behaviour, mental health and world view. Research has linked early traumatic life experiences in the parent’s childhood to disorganised attachment to their own child. In this paper we describe the data collected from parents enrolled in the Avon Longitudinal Study of Parents and Children (ALSPAC) on traumatic events experienced during their childhood, so that it can act as a resource for researchers in the future when considering outcomes on the adult, their children and grandchildren.

**Methods**: Data were collected via multiple questionnaires completed by parents enrolled into the ALSPAC study. During pregnancy and post-delivery, questionnaires were administered between 1990 and 1992 via post to the study mothers and their partners. Data were collected on life events including bereavement, sexual abuse, physical abuse, abandonment, neglect, memories of childhood and accidents. Other reports of traumatic events in childhood were reported by parents using free text. This can be made available to researchers for coding on request.

## Introduction

In creating the ALSPAC resource, the research team included questions designed to examine childhood experiences of trauma for the study mothers, their partners and the offspring. This paper focuses on the data collected on the childhoods of the mother and her partner.

Psychological trauma is a complex psychological state resulting from life events that are physically or emotionally harmful and have lasting adverse effects on wellbeing. It is believed that these effects can be passed down intergenerationally to the children of trauma sufferers in the form of relational trauma ^1^.

Traumatic events can include (but are not limited to) events such as war/conflict, childhood physical and sexual abuse, natural disasters, traumatic accidents, illness or witnessing events that result in death, threaten death, serious injury or a threat to the physical integrity of self or others ^2^. The symptoms resulting long after the trauma has occurred can include depression, anger, anxiety, dissociative disorders and posttraumatic stress disorder (PTSD) ^3^.

Whilst we were able to find a large amount of research into the effects of trauma during childhood, and into the effects on the children of parents who experience trauma in adulthood, there is much less research on the effects on the children of parents who have suffered trauma in childhood.

The research that has been carried out to date regarding the relationship between childhood trauma and the impact it has on the offspring of those who experience childhood trauma, is predominantly centred around PTSD caused by parental neglect, domestic or sexual abuse ^4, 5^ and war ^6, 7^.

However, there appear to be fewer research papers available regarding parental childhood trauma from other sources such as natural disasters ^8^, illness ^9^, extreme poverty ^10^, accidental injury ^11^, loss of a parent ^12^ or sibling, or being a witness to a distressing event ^13^ .

Fisher *et al.* (2013) ^14^, studied data from ALSPAC and found an association between adverse early life experiences, harsh parenting and bullying, and psychotic symptoms in the study children. The researchers used variables relating to the child’s lifestyle and life events as reported by the study mother, and evaluated the child’s early environment and bullying data, self-reported by the child to test for adversity. They then evaluated the child’s neurocognitive and psychological markers using these measures: Locus of Control gathered using a 12 item version of the Nowicki-Strickland Internal-External scale (NSIE) ^15^, self-esteem using a shortened form of Harter’s Self Perception Profile for Children ^16^, affective symptoms using the Development and Wellbeing Assessment (DAWBA) ^17^ and the Short Moods and Feelings Questionnaire (SMFQ) ^18 ^both completed by the child’s parents, and finally, 12 core questions from the semi-structured psychosis interview (PLIKSi) ^19^. Fisher *et al.* concluded that harsh parenting was not a factor for psychotic symptoms in the child but was indicated in depressive symptoms, anxiety, external locus of control and low self-esteem. It would be interesting to see whether the parent exhibiting the harsh behaviour also suffered from these symptoms as a result of similar or other traumatic experiences during their own childhoods and whether there is a link to intergenerational trauma here.

Plant *et al.* (2018) ^20^ analysed 9,397 ALSPAC mother child dyads with the aim of examining whether children whose mothers had a history of childhood trauma, were at increased risk of exhibiting psychopathology. The researchers tested three main hypotheses, that maternal child maltreatment (defined as whether the mother had been maltreated as a child) would predict child internalising and externalising difficulties, that maternal antenatal depression, maternal postnatal depression, maternal maladaptive parenting and child maltreatment would operate as independent mediators and finally, that there would be an indirect effect of maternal postnatal depression through maladaptive parenting and child maltreatment demonstrating separate cumulative effects. The researchers measured whether the mother had experienced physical abuse, sexual abuse, emotional abuse or neglect during their childhood (defined as <18 years). If the mother answered ‘yes’ to any of these exposures, they were considered to have experienced childhood maltreatment. They also measured whether the mothers showed signs of depression (antenatally and postnatally) using the Edinburgh Postnatal Depression Scale (EPDS) ^21^; and tested for symptoms of maladaptive parenting such as shouting, slapping and feelings of hostility towards their child. Child maltreatment (physical, sexual or emotional abuse) was elicited through maternal self-report. At 8 years of age the children were asked to self-report whether they had been bullied by their peers. The child was considered to have experienced maltreatment if a positive response had been given to any of these questions. Child depressive symptoms were measured at ages 10 and 13 by asking the mothers to complete the Development and Wellbeing Assessment (DAWBA) ^17^ and at 11 years, the Strengths and Difficulties (SDQ) ^22^ questionnaire. The researchers then looked at symptoms of attention-deficit hyperactivity disorder (ADHD), conduct disorder and oppositional defiant disorder (ODD), and found that maternal childhood maltreatment directly predicted the study child being exposed to maltreatment and subsequently developing psychopathology. This effect was greater when tested alongside maternal depression. Children of maltreated mothers had significantly greater emotional and behavioural difficulties at the ages of 10, 11 and 13, as well as greater peer conduct difficulties and hyperactivity problems. Maternal antenatal depression, postnatal depression and child maltreatment had an effect of independent mediation of the association between maternal child maltreatment and both internalising and externalising behaviour difficulties. Maladaptive parenting did not show this result.

 In this paper we describe the data collected from parents enrolled in the Avon Longitudinal Study of Parents and Children (ALSPAC) on traumatic events experienced during their childhood, so that it can act as a resource for researchers in the future when considering outcomes on the adult, their children and grandchildren.

## Methods

### Participants

14,541 pregnant mothers’ resident in the former Avon county of South West England were recruited into the ALSPAC study. These mothers all had an expected delivery date of 1 ^st^ April 1991 to 31 ^st^ December 1992. From these pregnancies, there were a total of 14,676 fetuses and 14,062 live births. Of these children, 13,988 were still alive at 1 year of age. Mothers were considered enrolled if they had returned at least one questionnaire or attended a “Children in Focus” clinic by 19 ^th^ July 1999. At the age of 7, the study team reached out to mothers who had previously not been included in the study and recruited additional families in order to boost the number of participants. As such, from the age of 7 the total sample number is 15,454 live births, resulting in 15,589 foetuses, of which 14,901 alive at 1 year of age ^23, 24^. However, these additional parents did not have data collected on their own childhood exposures.

In order to protect the confidentiality of the sample, data from triplet and quad multiple births have been removed as these children were considered to be at risk of identification. This leaves 14,691 eligible participants remaining. ALSPAC is continuing to monitor these families and are recruiting the Children of the Children of the 90’s ^25^. The ALSPAC team continue to gather data concerning the parents and grandparents of the study children, enabling further intergenerational research.

Following the advice of the ALSPAC Ethics and Law Committee, partners were recruited into the study only if the mothers wished them to be included. Questionnaires were sent to the mother who then passed the questionnaire on to the partner with a separate pre-paid return envelope. This method meant that ALSPAC were unable to follow up or communicate directly with the partners ^24, 26^. Therefore, the numbers of partners’ questionnaires returned were less than those received for the mother’s questionnaires. Around 75% of the partners participated in the study.

### Data collection

Since its inception, the ALSPAC study has collated thousands of variables from the study children and their families using questionnaires and clinics to collect the data.

This paper focuses on the variables we identified as being indicative of maternal childhood trauma experiences. There were 186 maternal variables which were collected from the A, B, C and D questionnaires, and 160 variables from the Partner’s collected via the PA, PB and PF questionnaires (Copies of these questionnaires can be viewed on the ALSPAC website). The variable naming convention for these questionnaires usually follows the format of the questionnaire’s assigned alphabetical letter followed by the number of the variable allocated in a sequential pattern in the data set.

The first four questionnaires were sent out at specific time points based on the mother’s gestation at enrolment. Partners were also surveyed at the mother’s discretion. The questionnaires were sent to the mother and the mother decided whether or not to pass that questionnaire to the partner.

Provided the mother enrolled before 14 weeks gestation, questionnaire A “Your Environment” and PA “You and Your Environment” were sent out on enrolment. If the mother enrolled after 14 weeks, the questionnaire was not sent until 22 weeks gestation. This was to prevent the mother feeling overwhelmed with questionnaires and to avoid clashes with the Having a Baby and Your Pregnancy questionnaires which were confined to certain gestation periods. In total, the Your Environment questionnaire was sent to 45% of participants before 15 weeks gestation, 32% between 22–25 weeks gestation and 23% were sent out later than 25 weeks.

Questionnaire B “Having a Baby” was sent out from 18 weeks gestation until 23 weeks gestation. If the mother enrolled before 24 weeks gestation, she also received the PB “Partners Questionnaire” alongside the B questionnaire. If the mother enrolled after 24 weeks, the PB “Partners Questionnaire” was sent out with the mothers “Your Home and Lifestyle” questionnaire. The “Your Home and Lifestyle” questionnaire is a version of the A “Your Environment” questionnaire which has been adapted for use by mothers who enrolled later into the study and therefore this data is coded alongside the A questionnaire data.

Questionnaire C “Your Pregnancy” was sent out from 32 weeks gestation until 40 weeks gestation. For questionnaire D “About Yourself” the timing of when this questionnaire was considered to be less important and therefore the bulk of these were sent out from 14 weeks gestation until 37 weeks gestation. However, in a small number of cases, questionnaire D was sent after the birth of the study child.

The H “Your Health Events and feelings” questionnaire was sent to mothers at 33 months postpartum and was accompanied by the PF “Partners Health Events and Feelings” questionnaire.

Please note that the study website contains details of all the data that is available through a fully searchable data dictionary and variable search tool.

The ALSPAC team have also provided a Questionnaire Topic guide which summarises the topics in each questionnaire.

The majority of the other questions available were drawn up by either the ALSPAC team or the European Longitudinal Study of Pregnancy and Childhood (ELSPAC) team. However, where suitable established measures existed, these measures were used within the questionnaire, occasionally with minor modifications.

### Sample demographics

Table 1a and Table 1b show the demographics of the parents who completed the questionnaires that contained the childhood events and circumstances. Most of these questionnaires (B, C, D, PA and PB) were completed during pregnancy, H and PF were administered 33 months after the birth of the child. The tables show the range of ages, education level, and ethnicity of the participating parents.

**Table 1a.** Demographic backgrounds of mothers who completed the B, C, D and H questionnaires.

	N	B Questionnaire	N	C Questionnaire	N	D Questionnaire	N	H Questionnaire
*Age of mother at* *birth of child (e695)* <25 25–34 35+	11,503	2,314 (20.12%) 7,978 (69.36%) 1,211 (10.53%)	11,307	2,224 (19.67%) 7,875 (69.65%) 1,208 (10.68%)	11,286	2,222 (19.69%) 7,870 (69.73%) 1,194 (10.58%)	9,287	1,597(17.20%) 6,649 (71.59%) 1,041 (11.21%)
*Mother’s highest* *education level* *(c645)* <O level O level >O level	11,447	2,863 (25.01%) 4,241 (37.05%) 4,343 (37.94%)	11,708	2,979 (25.44%) 4,324 (36.93%) 4,405 (37.62%)	11,168	2,750 (24.62%) 4,144 (37.11%) 4,274 (38.27%)	9,029	1,969 (21.81%) 3,347 (37.07%) 3,713 (41.12%)
*Partner lives with* *mother (a504*) Yes No	12,976	11,797 (90.91%) 1,179 (9.09%)	12,272	11,231 (91.52%) 1,041 (8.48%)	12,364	11,316 (91.52%) 1,048 (8.48%)	9,579	8,880 (92.70%) 699 (7.30%)
*Sex of child (kz021)* Male Female	13,269	6,853 (51.65%) 6,416 (48.35%)	12,546	6,480 (51.65%) 6,066 (48.35%)	12,535	6,450 (51.46%) 6,085 (48.54%)	9,738	5,008 (51.43%) 4,730 (48.57%)
*Mother’s Ethnic* *background (c800)* White Non-white	12,102	11,791 (97.43%) 311 (2.57%)	12,066	12,066 (97.37%) 326 (2.63%)	11,774	11,492 (97.60%) 282 (2.40%)	9,420	9,242 (98.11%) 178 (1.89%)

**Table 1b.** Demographic backgrounds of partners who completed the PA, PB and PF questionnaires.

	N	PA Questionnaire	N	PB Questionnaire	N	PF Questionnaire
*Age of partner at birth* *(pc996)* <25 25–34 35+	6,638	623 (9.39%) 4,500 (67.79%) 1,515 (22.82%)	7,138	707 (9.90%) 4,808 (67.36%) 1,623 (22.74%)	4,657	325 (6.98%) 3,171 (68.09%) 1,161 (24.93%)
*Partner’s highest education* *level (pb325)* <O level O level <O level	7,533	1,763 (23.40%) 1,820 (24.16%) 3,950 (52.44%)	9,333	2,357 (25.25%) 2,238 (23.98%) 4,738 (50.77%)	4,953	987 (19.93%) 1,157 (23.36%) 2,809 (56.71%)
*Partner lives with Mother* *(a504)* Yes No	8,616	8,219 (95.39%) 397 (4.61%)	9,857	9,355 (94.91%) 502 (5.09%)	5,456	5,267 (96.54%) 189 (3.46%)
*Sex of child (kz021)* Boy Girl	8,706	4,440 (51.00%) 4,266 (49.00%)	10,040	5,161 (51.40%) 4,879 (48.60%)	5,513	2,842 (51.55%) 2,671 (48.45%)
*Partner’s Ethnic background* *(pb440)* White Non-white	7,902	7,715 (97.63%) 187 (2.37%)	9,910	9,622 (97.09%) 288 (2.91%)	5,113	5,030 (98.38%) 83 (1.62%)

### Scale of life events

The life events questions to the mother ( Table 2a) comprised a set of 31 specific questions based on the previous work of Coddington ^27^, regarding events that may or may not have happened to the respondent before the age of 17. These questions were included in the questionnaire C sent to the mother at around 32 weeks gestation. 30 similar questions were also sent to the mother’s partners in their own questionnaire administered during pregnancy, the question to the mothers concerning whether they had acquired a stepsibling was inadvertently omitted from the partner’s questionnaire ( Table 2b). For each specified item the respondent was asked to choose between five possible answers: ‘ *yes, affected me a lot’*; ‘ *yes, moderately affected’*; ‘ *yes, mildly affected’*; ‘ *yes, but did not affect me’*; ‘ *no, did not happen’*. For each parent a question concerning anything else that occurred was asked, with a description written as text; these responses are not included here.

**Table 2a.** Mothers Life Events Scale indicating the potentially traumatic events that had occurred to the mother before 17 years of age, using questions based on Coddington ^4^ with the mother’s indication as to what effect they had had on her.

Variable Number	Question		Categorical responses					Binary responses	
		N	Yes, big effect	Yes, moderate effect	Yes, mild effect	Yes, no effect	No	Yes	No
	*Please indicate if any of the* *following events happened to* *you before you were 17 and how* *much it affected you*								
c400	Your parent died.	12,291	509 (4.14%)	110 (0.89%)	65 (0.53%)	45 (0.37%)	11,562 (94.07%)		
c400a	Your parent died.	12,291						729 (5.93%)	11,562 (94.07%)
c401	Your brother or sister died.	12,291	129 (1.05%)	54 (0.44%)	51 (0.41%)	65 (0.53%)	11,992 (97.57%)		
c401a	Your brother or sister died.	12,291						299 (2.43%)	11,992 (97.57%)
c402	A relative died.	12,291	878 (7.14%)	1,462 (11.89%)	2,452 (19.95%)	1,694 (13.78%)	5,805 (47.23%)		
c402a	A relative died.	12,291						6,486 (52.77%)	5.805 (47.23%)
c403	A friend died.	12,291	269 (2.19%)	476 (3.87%)	744 (6.05%)	244 (1.99%)	10,558 (85.90%)		
c403a	A friend died.	12,291						1,733 (14.10%)	10,558 (85.90%)
c404	A parent had a serious illness.	12,291	845 (6.87%)	628 (5.11%)	515 (4.19%)	198 (1.61%)	10,105 (82.21%)		
c404a	A parent had a serious illness.	12,291						2,186 (17.79%)	10,105 (82.21%)
c405	A parent was in hospital.	12,291	988 (8.04%)	1,033 (8.40%)	1,406 (11.44%)	1,314 (10.69%)	7,550 (61.43%)		
c405a	A parent was in hospital.	12,291						4,741 (38.57%)	7,550 (61.43%)
c406	You had a serious physical illness.	12,291	175 (1.42%)	152 (1.24%)	143 (1.16%)	120 (0.98%)	11,701 (95.20%)		
c406a	You had a serious physical illness.	12,291						590 (4.80%)	11,701 (95.20%)
c407	You were in hospital.	12,291	376 (3.06%)	621 (5.05%)	1,079 (8.78%)	1,971 (16.04%)	8,244 (67.07%)		
c407a	You were in hospital.	12,291						4,047 (32.93%)	8,244 (67.07%)
c408	Brother or sister had a serious illness.	12,291	203 (1.65%)	236 (1.92%)	277 (2.25%)	210 (1.71%)	11,365 (92.47%)		
c408a	Brother or sister had a serious illness.	12,291						926 (7.53%)	11,365 (92.47%)
c409	Brother or sister was in hospital.	12,291	244 (1.99%)	377 (3.07%)	721 (5.87%)	1,243 (10.11%)	9,706 (78.97%)		
c409a	Brother or sister was in hospital.	12,291						2,585 (21.03%)	9,706 (78.97%)
c410	A parent had a serious accident.	12,291	141 (1.15%)	127 (1.03%)	139 (1.13%)	100 (0.81%)	11,784 (95.88%)		
c410a	A parent had a serious accident.	12,291						507 (4.12%)	11,784 (95.88%)
c411	You had a serious accident.	12,291	98 (0.80%)	104 (0.85%)	145 (1.18%)	94 (0.76%)	11,850 (96.41%)		
c411a	You had a serious accident.	12,291						441 (3.59%)	11,850 (96.41%)
c412	Brother or sister had a serious accident.	12,291	132 (1.07%)	143 (1.16%)	170 (1.38%)	116 (0.94%)	11,730 (95.44%)		
c412a	Brother or sister had a serious accident.	12,291						561 (4.56%)	11,730 (95.44%)
c413	You acquired a physical deformity.	12,291	34 (0.28%)	20 (0.16%)	25 (0.20%)	15 (0.12%)	12,197 (99.24%)		
c413a	You acquired a physical deformity.	12,291						94 (0.76%)	12,197 (99.24)
c414	You became pregnant.	12,291	454 (3.69%)	152 (1.24%)	140 (1.14%)	177 (1.44%)	11,368 (92.49%)		
c414a	You became pregnant.	12,291						923 (7.51%)	11,368 (92.49%)
c415	A parent was imprisoned.	12,291	24 (0.20%)	15 (0.12%)	20 (0.16%)	33 (0.27)	12,199 (99.25)		
c415a	A parent was imprisoned.	12,291						92 (0.75%)	12,199 (99.25%)
c416	A parent was physically cruel to you.	12,291	183 (1.49%)	120 (0.98%)	84 (0.68%)	34 (0.28%)	11,870 (96.57%)		
c416a	A parent was physically cruel to you.	12,291						421 (3.43%)	11,870 (96.57%)
c417	Your parents separated.	12,291	829 (6.74%)	570 (4.64%)	398 (3.24%)	300 (2.44%)	10,194 (82.94%)		
c417a	Your parents separated.	12,291						2,097 (17.06%)	10,194 (82.94%)
c418	Your parents divorced.	12,291	680 (5.53%)	496 (4.04%)	377 (3.07%)	342 (2.78%)	10,396 (84.58%)		
c418a	Your parents divorced.	12,291						1,895 (15.42%)	10,396 (84.58%)
c419	A parent remarried.	12,291	367 (2.99%)	336 (2.73%)	306 (2.49%)	422 (3.43%)	10,860 (88.36%)		
c419a	A parent remarried.	12,291						1,431 (11.64%)	10,860 (88.36%)
c420	A parent was emotionally cruel to you.	12,291	390 (3.17%)	264 (2.15%)	231 (1.88%)	53 (0.43%)	11,353 (92.37%)		
c420a	A parent was emotionally cruel to you.	12,291						938 (7.63%)	11,353 (92.37%)
c421	Your parents had serious arguments.	12,291	843 (6.86%)	883 (7.18%)	1,060 (8.62%)	506 (4.12%)	8,999 (73.22%)		
c421a	Your parents had serious arguments.	12,291						3,292 (26.78%)	8,999 (73.22%)
c422	You were sexually abused.	12,291	368 (2.99%)	131 (1.07%)	93 (0.76%)	36 (0.29%)	11,663 (94.89%)		
c422a	You were sexually abused.	12,291						628 (5.11%)	11,663 (94.89%)
c423	A parent was mentally ill.	12,291	167 (1.36%)	145 (1.18%)	142 (1.16%)	63 (0.51%)	11,774 (95.79%)		
c423a	A parent was mentally ill.	12,291						517 (4.21%)	11,774 (95.79%)
c424	You discovered you were adopted.	12,291	48 (0.39%)	49 (0.40%)	43 (0.35%)	111 (0.90%)	12,040 (97.96%)		
c424a	You discovered you were adopted.	12,291						251 (2.04%)	12,040 (97.96%)
									
c425	Your family moved to a new district.	12,291	461 (3.75%)	667 (5.43%)	900 (7.32%)	1,288 (10.48%)	8,975 (73.02%)		
c425a	Your family moved to a new district.	12,291						3,316 (26.98%)	8,975 (73.02%)
c426	You were in trouble with the police.	12,291	61 (0.50%)	83 (0.68%)	172 (1.40%)	177 (1.44%)	11,798 (95.99%)		
c426a	You were in trouble with the police.	12,291						493 (4.01%)	11,798 (95.99%)
c427	You were expelled or suspended from school.	12,291	38 (0.31%)	55 (0.45%)	134 (1.09%)	226 (1.84%)	11,838 (96.31%)		
c427a	You were expelled or suspended from school.	12,291						453 (3.69%)	11,838 (96.31%)
c428	You failed an important exam.	12,291	159 (1.29%)	338 (2.75%)	562 (4.57%)	451 (3.67%)	10,781 (87.71%)		
c428a	You failed an important exam.	12,291						1,510 (12.29%)	10,781 (87.71%)
c429	Your family’s financial circumstances got worse.	12,291	172 (1.40%)	330 (2.68%)	565 (4.60%)	479 (3.90%)	10,745 (87.42%)		
c429a	Your family’s financial circumstances got worse.	12,291						1,546 (12.58%)	10,745 (87.42%)
c430	You acquired a stepbrother or stepsister.	12,291	128 (1.04%)	133 (1.08%)	232 (1.89%)	456 (3.71%)	11,342 (92.28%)		
c430a	You acquired a stepbrother or stepsister.	12,291						949 (7.72%)	11,342 (92.28%)
c431	Other important happening.	12,291	224 (1.82%)	59 (0.48%)	22 (0.18%)	9 (0.07%)	11,977 (97.45%)		
c431a	Other important happening.	12,293						313 (2.57%)	11,880 (97.43%)

**Table 2b.** Partner’s Life Events Scale indicating the potentially traumatic events that had occurred to the mother’ before 17 years of age, using questions based on Coddington ^4^ with the mother’s partner’s indication as to what effect they had had on him.

Variable Number	Question		Categorical Responses					Binary responses	
		N	Affected a lot	Affected a bit	Mild effect	No effect	Did not happen	Yes	No
	*Please indicate if any of the* *following events happened to you* *before you were 17 and how much* *it affected you.*								
pb450	A parent died	9,957	409 (4.11%)	101 (1.01%)	42 (0.42%)	55 (0.55%)	9,350 (93.90%)		
pb450a	A parent died	9,957						607 (6.10%)	9,350 (93.90%)
pb451	A brother or sister died	9,957	112 (1.12%)	46 (0.46%)	51 (0.51%)	134 (1.35%)	9,614 (96.56%)		
pb451a	A brother or sister died	9,957						343 (3.44%)	9,614 (96.56%)
pb452	A relative died	9,957	693 (6.96%)	1,038 (10.42%)	2,204 (22.14%)	2,171 (21.80%)	3,851 (38.68%)		
pb452a	A relative died	9,957						6,106 (61.32%)	3,851 (38.68%)
pb453	A friend died	9,957	273 (2.74%)	508 (5.10%)	790 (7.93%)	567 (5.69%)	7,819 (78.53%)		
pb453a	A friend died	9,957						2,138 (21.47%)	7,819 (78.53%)
pb454	A parent had a serious illness	9,957	739 (7.42%)	614 (6.17%0	556 (5.58%)	374 (3.76%)	7,674 (77.07%)		
pb454a	A parent had a serious illness	9,957						2,283 (22.93%)	7,674 (77.07%)
pb455	A parent was in hospital	9,957	748 (7.51%)	808 (8.11%)	1,218 (12.23%)	1,332 (13.38%)	5,851 (58.76%)		
pb455a	A parent was in hospital	9,957						4,106 (41.24%)	5,851 (58.76%)
pb456	You had a serious physical illness	9,957	333 (3.34%)	194 (1.95%)	277 (2.78%)	249 (2.50%)	8,904 (89.42%)		
pb456a	You had a serious physical illness	9,957						1,053 (10.58%)	8,904 (89.42%)
pb457	You were in hospital	9,957	409 (4.11%)	475 (4.77%)	938 (9.42%)	2,131 (21.40%)	6,004 (60.30%)		
pb457a	You were in hospital	9,957						3,953 (39.70%)	6,004 (60.30%)
pb458	Brother or sister had a serious illness	9,957	174 (1.75%)	197 (1.98%)	321 (3.22%)	371 (3.73%)	8,894 (89.32%)		
pb458a	Brother or sister had a serious illness	9,957						1,063 (10.68%)	8,894 (89.32%)
pb459	Brother or sister was in hospital	9,957	213 (2.14%)	269 (2.70%)	568 (5.70%)	1,343 (13.49%)	7,564 (75.97%)		
Pb459a	Brother or sister was in hospital	9,957						2,393 (24.03%)	7,564 (75.97%)
pb460	A parent had a serious accident		9,957	143 (1.44%)	138 (1.39%)	183 (1.84%)	172 (1.73%)	9,321 (93.61%)	
pb460a	A parent had a serious accident	9,957						636 (6.39%)	9,321 (93.61%)
pb461	You had a serious accident	9,957	209 (2.09%)	183 (1.84%)	240 (2.41%)	304 (3.05%)	9,022 (90.61%)		
pb461a	You had a serious accident	9,957						935 (9.39%)	9,022 (90.61%)
pb462	Brother or sister had a serious accident	9,957	116 (1.17%)	140 (1.41%)	216 (2.17%)	217 (2.18%)	9,268 (93.08%)		
pb462a	Brother or sister had a serious accident	9,957						689 (6.92%)	9,268 (93.08%)
pb463	You acquired a physical deformity	9,957	41 (0.41%)	36 (0.36%)	39 (0.39%)	39 (0.39%)	9,802 (98.44%)		
pb463a	You acquired a physical deformity	9,957						155 (1.56%)	9,802 (98.44%)
pb464	Your girlfriend became pregnant	9,957	152 (1.53%)	53 (0.53%)	57 (0.57%)	67 (0.67%)	9,628 (96.70%)		
pb464a	Your girlfriend became pregnant	9,957						329 (3.30%)	9,628 (96.70%)
pb465	A parent was imprisoned	9,957	38 (0.38%)	16 (0.16%)	23 (0.23%)	60 (0.60%)	9,820 (98.62%)		
pb465a	A parent was imprisoned	9,957						137 (1.38%)	9,820 (98.62%)
pb466	A parent was physically cruel to you	9,957	171 (1.72%)	93 (0.93%)	128 (1.29%)	99 (0.99%)	9,466 (95.07%)		
pb466a	A parent was physically cruel to you	9,957						491 (4.93%)	9,466 (95.07%)
pb467	Your parents separated	9,957	641 (6.44%)	339 (3.40%)	313 (3.14%)	261 (2.62%)	8,403 (84.39%)		
pb467a	Your parents separated	9,957						1,554 (15.61%)	8,403 (84.39%)
pb468	Your parents divorced	9,957	461 (4.63%)	268 (2.69%)	256 (2.57%)	277 (2.78%)	8,695 (87.33%)		
pb468a	Your parents divorced	9,957						1,262 (12.67%)	8,695 (87.33%)
pb469	A parent remarried	9,957	245 (2.46%)	183 (1.84%)	226 (2.27%)	336 (3.37%)	8,967 (90.06%)		
pb469a	A parent remarried	9,957						990 (9.94%)	8,967 (90.06%)
pb470	A parent was emotionally cruel to you	9,957	247 (2.48%)	153 (1.54%)	212 (2.13%)	87 (0.87%)	9,258 (92.98%)		
pb470a	A parent was emotionally cruel to you	9,957						699 (7.02%)	9,258 (92.98%)
pb471	Your parents had serious arguments	9,957	665 (6.68%)	664 (6.67%)	942 (9.46%)	815 (8.19%)	6,871 (69.01%)		
pb471a	Your parents had serious arguments	9,957						3,086 (30.99%)	6,871 (69.01%)
pb472	You were sexually abused	9,957	53 (0.53%)	24 (0.24%)	23 (0.23%)	23 (0.23%)	9,834 (98.76%)		
pb472a	You were sexually abused	9,957						123 (1.24%)	9,834 (98.76%)
pb473	A parent was mentally ill	9,957	123 (1.24%)	85 (0.85%)	67 (0.67%)	76 (0.76%)	9,606 (96.47%)		
pb473a	A parent was mentally ill	9,957						351 (3.53%)	9,606 (96.47%)
pb474	You discovered you were adopted	9,957	43 (0.43%)	29 (0.29%)	38 (0.38%)	92 (0.92%)	9,755 (97.97%)		
pb474a	You discovered you were adopted	9,957						202 (2.03%)	9,755 (97.97%)
pb475	Your family moved to a new district	9,957	395 (3.97%)	501 (5.03%)	853 (8.57%)	1,423 (14.29%)	6,785 (68.14%)		
pb475a	Your family moved to a new district	9,957						3,172 (31.86%)	6,785 (68.14%)
pb476	You were in trouble with the police	9,957	263 (2.64%)	245 (2.46%)	524 (5.26%)	926 (9.30%)	7,999 (80.34%)		
pb476a	You were in trouble with the police	9,957						1,958 (19.66%)	7,999 (80.34%)
pb477	You were expelled or suspended from school	9,957	93 (0.93%)	82 (0.82%0	220 (2.21%)	610 (6.13%0	8,952 (89.91%)		
pb477a	You were expelled or suspended from school	9,957						1,005 (10.09%)	8,952 (89.91%)
pb478	You failed an important exam	9,957	210 (2.11%)	316 (3.17%)	646 (6.49%)	978 (9.82%)	7,807 (78.41%)		
pb478a	You failed an important exam	9,957						2,150 (21.59%)	7,807 (78.41%)
pb479	Your family’s financial circumstances got worse	9,957	188 (1.89%)	272 (2.73%)	447 (4.49%)	606 (6.09%)	8,444 (87.80%)		
pb479a	Your family’s financial circumstances got worse	9,957						1,513 (15.20%)	8,444 (84.80%)
pb480	Another important happening (please tick and describe)	9,957	186 (1.87%)	46 (0.46%)	31 (0.31%)	16 (0.16%)	9,678 (97.20%)		
pb480a	Another important happening (please tick and describe)	9,957						279 (2.80%)	9,678 (97.20%)

**Figure 1.**
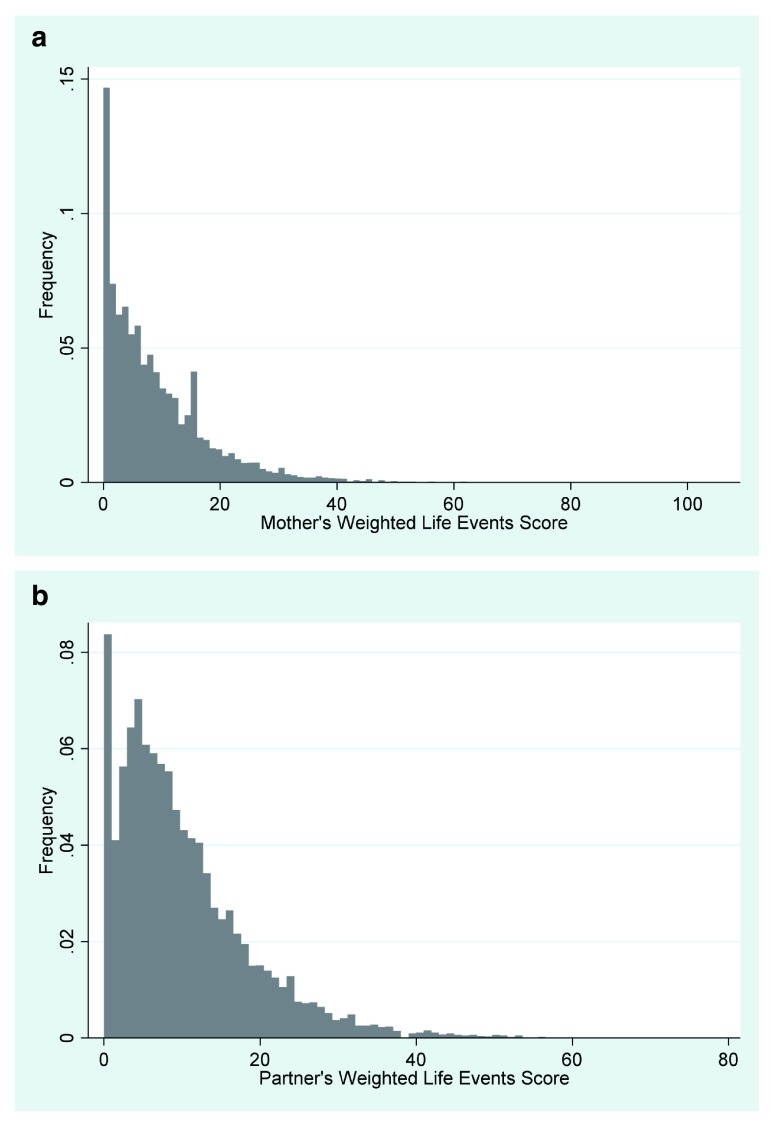
**a**. Mother’s Weighted Life Events Score. **b**. Partner’s Weighted Life Events Score.

From the life events scale, two variables were created. The first, the *weighted life events scores* (c432 and pb481) gives an indication of the perceived degree of effect of each life event the parent experienced. Thus, c432 was created by selecting variables c400-c430, reversing the coding for these variables and setting ‘no’ to 0 (unless all were missing in which case the variable was left as missing). Therefore, greater perceived effects will have a higher score; addition of all life events variables together provides the overall score. [Variable c431 was not included in the score as analysis showed that when another event was noted, it closely matched another variable between c400-c430 and had been coded into that variable]. The same coding system was used for the partners’ variables, pb450 to pb479 ( Figure 1a, Figure1b). The range for mothers and partners were 0-107 and 0-78, with medians of 6 and 7 respectively.

**Figure 2.**
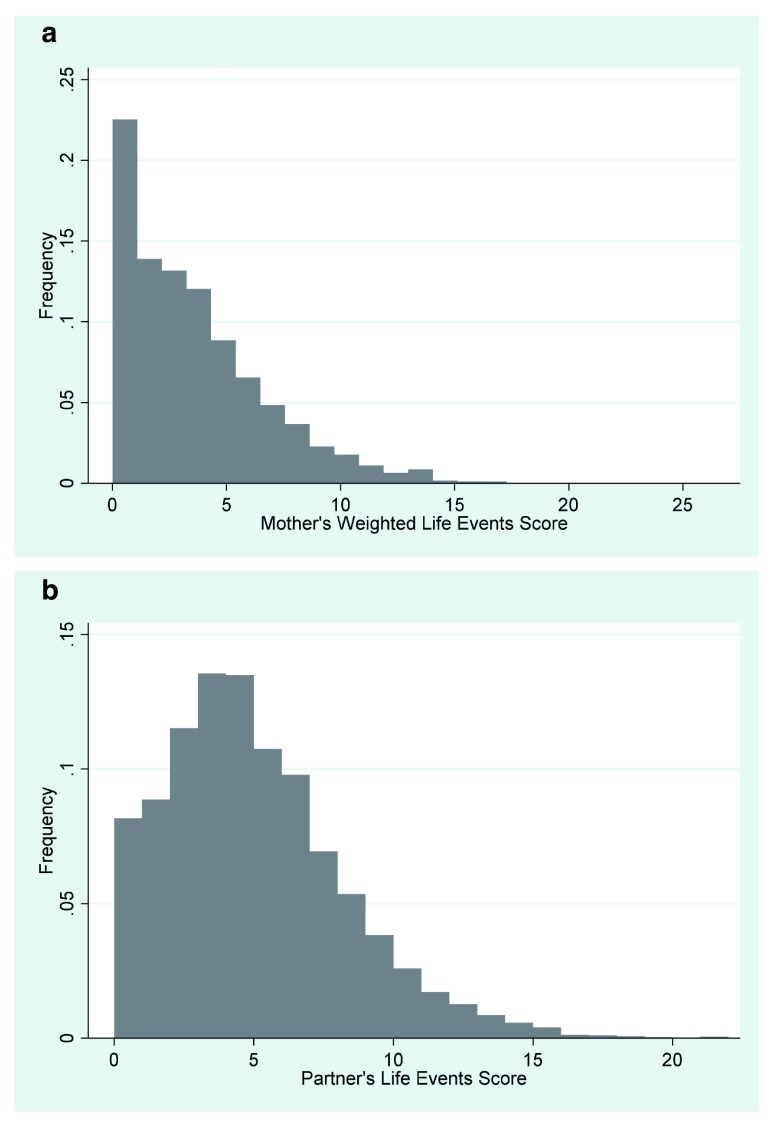
**a**. Mother’s Life Events Score. **b**. Partner’s Life Events Score.

The second derived variables, the Life Events Scores (c433 and pb482), gives the number of life events experienced by each parent and was created by recoding the variables to make all ‘yes’ responses as 1 and the ‘no’ responses as 0, and then adding these variables together. For the mother’s data, the variables used to create the score were c400-c430. The variables used to create the partners’ score were pb450 to pb479. The ranges were 0-27 and 0-22 and medians 3 and 3.5 for mothers and partners respectively ( Figures 2a, 2b).

### Other childhood circumstances

Questions relating to other circumstances during the parents’ childhood can be found in Table 3a and Table 3b. Variables indicating further questions where the respondent was asked to indicate their actual age at which circumstances occurred are shown in Table 4a and Table 4b. Although not listed in this table, ages are also available for when specific accidents and injuries had occurred, thus enabling childhood events to be computed (see variables d080 to d130 for the mothers and pa110 to pa163 for their partners).

**Table 3a.** Mother’s childhood circumstances that might indicate stress during childhood as self-reported in the D questionnaire.

Variable Number	Question		Categorical responses					Binary responses		
		**N**	**Yes**	**No**	**Unsure**					
d380 *	*Were you legally adopted?*	12,293	354 (2.88%)	11,939 (97.12%)						
d385	*Were you ever “in care” of either a* *local authority or voluntary agency* *e.g. Barnardo’s?*	11,911	264 (2.22%)	11,564 (97.09%)	83 (0.70%)					
d387	*Did your parents’ divorce or* *separate before your 18 ^th^ birthday?*	12,235	2,372 (19.39%)	9,863 (80.61%)						
		**N**	**Mother**	**Father**	**Some-times Mum, Some-times Dad**	**Someone else**				
d389	*Who did you mainly live with after parents* *divorced or separated?*	2,328	1,757 (75.47%)	315 (13.53%)	88 (3.78%)	168 (7.22%)				
			**Yes**	**No**						
	*Did you ever live away from home* *with any of the following (other than* *holidays or short visits) before* *you were 18 years old?*	**N**	**Yes**	**No**	**Don’t Know**					
d392	Grandparents	12,294	654 (5.32%)	11,640 (94.68%)						
d393	Other relatives	12,294	493 (4.01%)	11,801 (95.99%)						
d394	Friends	12,294	469 (3.81%)	11,825 (96.19%)						
d395	Foster parents	12,294	161 (1.31%)	12,132 (98.68%)	<5 (0.01%)					
d396	Other home	12,294	641 (5.21%)	11,653 (94.79%)						
		**N**	**Yes <1 week**	**Yes 1 week to 1month**	**Yes 1 to 6 months**	**Yes >6 months**	**Yes, Not known how long**	**No**	**Yes**	**No**
	*Did you ever stay away from home* *in any of the following places before* *you were 18 years old?*									
d400	Hospital	12,250	3,085 (25.18%)	1,654 (13.50%)	203 (1.66%)	34 (0.28%)		7,274 (59.38%)		
d400a	Hospital	12,250							4,976 (40.62%)	7,274 (59.38%)
d401	Boarding school	12,198	15 (0.12%)	24 (0.20%)	65 (0.53%)	359 (2.94%)		11,735 (96.20%)		
d401a	Boarding school	12,198							463 (3.80%)	11,735 (96.20%)
d402	Children’s home	12,190	6 (0.05%)	21 (0.17%)	45 (0.37%)	121 (0.99%)		11,997 (98.42%)		
d402a	Children’s home	12,190							193 (1.58%)	11,997 (98.42%)
d403	Hostel	12,148	74 (0.61%)	69 (0.57%)	20 (0.16%)	37 (0.30%)		11,948 (98.35%)		
d403a	Hostel	12,148							200 (1.65%)	11,948 (98.35%)
d404	Stayed in custody	12,136	<5 (0.03%)	7 (0.06%)	10 (0.08%)	<5 (0.03%)		12,111 (99.79%)		
d404a	Stayed in custody	12,136							25 (0.21%)	12,111 (99.79%)
d405	Stayed in another place	12,360	84 (0.68%)	275 (2.22%)	111 (0.90%)	211 (1.71%)	16 (0.13%)	11,663 (94.36%)		
d405s	Stayed in another place	12,360							697 (5.64%)	11,663 (94.36%)
d410	Did you leave home before your 18 ^th^ birthday?	12,299							2,451 (19.93%)	9,848 (80.07%)
		**N**	**College accommodation**	**Hostel**	**Bedsit**	**Shared accommodation**		**Other**	**Yes**	**No**
d411	*Where Mother lived when she first* *left home before 18 years*	2,336	232 (9.93%)	47 (2.01%)	413 (17.68%)	864 (36.99%)		780 (33.39%)		
	*Before you were 17 did a parent or* *person who cared for you die?*								**Yes**	**No**
d620	Mother	12,448							218 (1.75%)	12,230 (98.25%)
d621	Father	12,448							491 (3.94%)	11,957 (96.06%)
d622	Mother figure	12,448							33 (0.27%)	12,415 (99.73%)
d623	Father figure	12,448							37 (0.30%)	12,411 (99.70%)
d624	Other (please describe)	12,448							591 (4.75%)	11,857 (95.25%)
d625	*Carer died before Mum was 17*	12,448							1,289 (10.36%)	11,159 (89.64%)
		**N**	**Always**	**Mostly**	**Rarely**	**Never**				
	*Was your parent’s behaviour stable* *and predictable to you as a child?*									
d750	Mother	12,027	6,833 (56.81%)	4,543 (37.77%)	470 (3.91%)	181 (1.50%)				
d751	Father	11,465	6,281 (54.78%)	4,168 (36.35%)	711 (6.20%)	305 (2.66%)				
d752	Mother figure	436	202 (46.33%)	157 (36.01%)	38 (8.72%)	39 (8.94%)				
d753	Father figure	697	288 (41.32%)	274 (39.31%)	73 (10.47%)	62 (8.90%)				
		**N**	**Very stable**	**Fairly stable**	**Unstable**	**Very unstable**				
d755	*Stability of home in childhood*	12,230	5,561 (45.47%)	5,139 (42.02%)	1,054 (8.62%)	476 (3.89%)				

* *d380 spans ages beyond 17 so only cases where the event happened before 18 years of age have been coded at “yes”. The full age ranges available can be found in Table 4a*

**Table 3b.** Partner’s childhood circumstances that might indicate stress during childhood as self-reported in the PA questionnaire

Variable Number	Question		Categorical responses						Binary responses	
		*N*	Yes	No	Unsure				Yes	No
pa380*	*Were you legally adopted?*	8,405							199 (2.37%)	8,206 (97.63%)
pa385	*Were you ever in care of either* *a local authority or voluntary agency* *Barnardo’s?*	8,162	176 (2.16%)	7,926 (97.11%)	60 (0.74%)					
pa387	*Did your parents’ divorce or* *separate before your 18 ^th^ birthday?*	8,330							1,312 (15.75%)	7,018 (84.25%)
		**N**	**Mother**	**Father**	**Shared** **Mother** **and Father**	**Someone else**			**Yes**	**No**
pa389	*Who did you live with after parents* *divorced?*	8,317	891 (69.12%)	214 (16.60%)	59 (4.58%)	125 (9.70%)				
	*Did you ever live away from home* *with any of the following (other than* *for holidays/ or short visits) before* *you were 18 years old?*									
pa392	Grandparents	8,345							481 (5.76%)	7,864 (94.24%)
pa393	Other relatives	8,345							342 (4.10%)	8,003 (95.90%)
pa394	Friends	8,345							303 (3.63%)	8,042 (96.37%)
pa395	Foster parents	8,345							71 (0.85%)	8,274 (99.15%)
pa396	Other	8,345							517 (6.20%)	7,828 (93.80%)
		**N**	**Yes, <1 week**	**Yes, 1** **week to** **1 month**	**Yes, 1 to 6** **months**	**Yes, > 6 months**	**Yes,** **duration** **not known**	**No, did not** **happen**	**Yes**	**No**
	*Did you ever stay away from home* *in any of the following places before* *you were 18 years old?*									
pa400	Hospital	8,268	2,106 (25.47%)	1,399 (16.92%)	273 (3.30%)	66 (0.80%)		4,424 (53.51%)		
pa400a	Hospital	8,268							3,844 (46.49%)	4,424 (46.49%)
pa401	Boarding school	8,238	5 (0.06%)	23 (0.28%)	65 (0.79%)	436 (5.29%)		7,709 (93.58%)		
pa401a	Boarding school	8,238							529 (6.42%)	7,709 (93.58%)
pa402	Children’s home	8,212	<5 (0.02%)	21 (0.26%)	19 (0.23%)	90 (1.10%)		8,080 (98.39%)		
pa402a	Children’s home	8,212							132 (1.61%)	8,080 (98.39%)
pa403	Hostel	8,186	96 (1.17%)	122 (1.49%)	11 (0.13%)	30 (0.37%)		7,927 (96.84%)		
pa403a	Hostel	8,186							259 (3.16%)	7,927 (96.84%)
pa404	Stayed in custody	8,185	45 (0.55%)	31 (0.38%)	58 (0.71%)	67 (0.82%)		7984 (97.54%)		
pa404a	Stayed in custody	8,185							201 (2.46%)	7,984 (97.54%)
pa405	Other	8,410	65 (0.77%)	263 (3.13%)	82 (0.98%)	244 (2.90%)	41 (0.49%)	7,715 (91.74%)		
pa405a	Other	8,410							695 (8.26%)	7,715 (91.74%)
pa410	*Did you leave home before your* *18 ^th^ birthday?*	8,300							1,561 (18.81%)	6,739 (81.19%)
		**N**	**College Accommodation**	**Hostel**	**Bedsit**	**Shared Accommodation**	**Other**			
pa411	*Where partner lived when they left* *home <18 years*	1,498	261 (17.42%)	37 (2.47%)	233 (15.55%)	405 (27.04%)	562 (37.52%)			
			**Yes, both**	**Yes, mother**	**Yes, father**	**No, neither**			**Yes**	**No**
	*Before you were 17 did a parent or* *person who cared for you die?*									
pa619	Either parent died	1,191	<5 (0.08%)	16 (1.34%)	58 (4.87%)	1,116 (93.70%)				
pa620	Mother	8,621							116 (1.35%)	8,505 (98.65%)
pa621	Father	8,621							283 (3.28%)	8,338 (96.72%)
pa622	Mother figure	8,621							11 (0.13%)	8,610 (99.87%)
pa623	Father figure	8,621							24 (0.28%)	8,597 (99.72%)
pa624	Other	8,621							390 (4.52%)	8,231 (95.48%)
pa625	One of above carers died	8,621							789 (9.15%)	7,832 (90.85%)
			**Yes,** **hospitalised**	**Yes, saw** **a doctor**	**Yes, home** **treatment**	**No, never**			**Yes**	**No**
	*Have any of the following ever happened?*									
pa131*	You were sexually assaulted	8,371	N/A	13 (0.16%)	66 (0.79%)	8,292 (99.06%)				
pa132*	You were sexually assaulted	8,371							79 (0.69%)	8,313 (99.31%)
		**N**	**Always**	**Mostly**	**Rarely**	**Never**				
	*Was your parent’s behaviour stable* *and predictable as a child?*									
pa750	Mother	8,161	4,715 (57.77%)	3,113 (38.14%)	232 (2.84%)	101 (1.24%)				
pa751	Father	7,809	4,288 (54.91%)	2,953 (37.82%)	399 (5.11%)	169 (2.16%)				
pa752	Mother figure	303	147 (48.51%)	118 (38.94%)	17 (5.61%)	21 (6.93%)				
pa753	Father figure	453	209 (46.14%)	173 (38.19%)	38 (8.39%)	33 (7.28%)				
		**N**	**Very stable**	**Fairly stable**	**Unstable**	**Very unstable**				
pa755	*Home stability in childhood*	8,296	3,892 (46.91%)	3,607 (43.48%)	563 (6.79%)	234 (2.82%)				

* pa131, pa132and pa380 span ages beyond 17 so cases where the event happened after 18 years of age have been coded as “no”. The full age ranges available for these events can be found in Table 4b in variables pa133 and pa381

**Table 4a.** Questions asked concerning the mother’s notable events together with for her age at which the event occurred, thus enabling the women who had experienced the event at particular stages of childhood to be identified.

Variable Number	Question	N	Range
b023	*Age when pregnant for the very first time?*	13,179	10 – 44
d103	*Age Mother was sexually assaulted*	418	2 – 34
d381	*Age Mother was adopted*	318	0 – 27
d388	*Age when her parents divorced or* *separated before 18 ^th^ birthday*	2,211	0 – 17
d630	*Age when her mother died*	408	0 – 17+
d631	*Age her father died*	789	0 – 17+
d632	*Age when her Mother figure died*	55	0 – 17+
d633	*Age when Father figure died*	59	0 – 17+
d634	*Age when another carer died*	576	0 – 17+
h136d	*Age when first physically abused*	458	0 – 16

**Table 4b.** Questions asked concerning the notable events occurring to the mother’s partner during childhood, together with his age at which the event occurred, thus enabling the partner who had experienced the event at particular stages of childhood to be identified.

Variable number	*Question*	*N*	*Range*
pa133	*Age when he was sexually assaulted*	71	1–33
pa381	*Age he was legally adopted*	207	0–40
pa388	*Age when his parents divorced or separated*	1,213	0–18+
pa630	*Age when his mother died*	339	0–17+
pa631	*Age when his father died*	675	0–17+
pa632	*Age when his mother figure died*	71	0–17+
pa633	*Age when his father figure died*	88	0–17+
pa634	*Age when another carer died*	399	0–17+

Subsequent to pregnancy, at 33 months after delivery, further questions were asked about abuse during childhood as well as of violent events in the home. The reason for the delay in asking these questions concerned the realisation that they should have been asked earlier but would still be valid to ask at this stage in the parents’ life course ( Table 5a and Table 5b).

**Table 5a.** Mothers recall of childhood abuse and domestic violence asked at 33 months postpartum.

Variable Number	Question		Categorical responses				Binary responses	
		**N**	**Yes, Severely**	**Yes, somewhat**	**No, not at all**			
h134	*Did you feel emotionally neglected* *during your childhood?*	9,574	251 (2.62%)	1,844 (19.26%)	7,479 (78.12%)			
h135	*Were you physically neglected* *as a child (e.g.* *not fed or clothed properly)?*	9,585	25 (0.26%)	165 (1.72%)	9,395 (98.02%)			
h136	*Were you physically abused (e.g. beaten) as a child?*	9,553	71 (0.74%)	525 (5.50%)	8,957 (93.76%)			
	*Who abused you?*						**Yes**	**No**
h136a	Mother	9,361					248 (2.65%)	9,113 (97.35%)
h136b	Father	9,393					326 (3.47%)	9,067 (96.53%)
h136c	Another person	9,133					176 (1.93%)	8,957 (98.07%)
		**N**	**Yes, always**	**Yes, frequently**	**Yes, some-what**	**No, Not at all**		
	*How would you describe the* *relationship between your mother* *and father when you were growing up?*							
h137a	Violent	8,819	59 (0.67%)	185 (2.10%)	949 (10.76%)	7,626 (86.47%)		
h137b	Affectionate	8,934	1,367 (15.30%)	2,273 (25.44%)	4,178 (46.77%)	1,116 (12.49%)		
h137c	Quarrelsome	8,993	330 (3.67%)	1,524 (16.95%)	5,139 (57.11%)	2,003 (22.27%)		
h137d	Happy	9,002	2,107 (23.41%)	3,716 (41.28%)	2,718 (30.19%)	461 (5.12%)		
h137e	Frightening	8,880	77 (0.87%)	274 (3.09%)	1,355 (15.26%)	7,174 (80.79%)		
h137f	Friendly	8,938	3,293 (36.84%)	3,163 (35.39%)	2,151 (24.07%)	331 (3.70%)		
h137g	Respectful	8,905	3,260 (36.61%)	2,617 (29.39%)	2,236 (25.11%)	792 (8.89%)		
h137h	Remote	8,863	246 (2.78%)	696 (7.85%)	2,797 (31.56%)	5,124 (57.81%)		

**Table 5b.** Mothers’ partners’ recall of childhood abuse and domestic violence asked at 33 months postpartum.

Variable Number	Question	Categorical Responses						Binary responses	
		**N**	**Yes,** **Severely** **Neglected**	**Yes,** **Somewhat** **Neglected**	**No, not at** **all**				
pf2060	*Did you feel neglected* *emotionally during your* *childhood?*	5,412	106 (1.96%)	976 (18.03%)	4,330 (80.01%)				
pf2061	*Were you physically neglected* *as a child (e.g. not fed or* *clothed properly)?*	5,425	22 (0.41%)	117 (2.16%)	5,286 (97.44%)				
		**N**	**Yes,** **Severely** **Abused**	**Yes,** **Somewhat** **Abused**	**No, not at** **all**			**Yes**	**No**
pf2062	*Were you physically abused* *(e.g. beaten) as a child?*	5,422	40 (0.74%)	274 (5.05%)	5,108 (94.21%)				
	*Who abused you?*								
pf2063	Mother	290						104 (35.86%)	186 (64.14%)
pf2064	Father	328						220 (67.07%)	108 (32.93%)
pf2065	Someone else	79						79 ( *%)	- *
		**N**	**Yes,** **always**	**Yes,** **frequently**	**Yes,** **Sometimes**	**No, Not** **at all**	**Single** **parent** **family**		
	*How would you describe the* *relationship between your* *mother and father when you* *were growing up?*								
pf2070	Violent	4,771	14 (0.29%)	100 (2.10%)	475 (9.96%)	4,182 (87.65%)			
pf2071	Affectionate	4,921	630 (12.80%)	1,295 (26.32%)	2,414 (49.06%)	457 (9.29%)	125 (2.54%)		
pf2072	Quarrelsome	4,831	93 (1.93%)	681 (14.10%)	2,826 (58.50%)	1,231 (25.48%)			
pf2073	Happy	4,852	1,030 (21.23%)	2,212 (45.59%)	1,435 (29.58%)	175 (3.61%)			
pf2074	Frightening	4,787	27 (0.56%)	101 (2.11%)	673 (14.06%)	3,986 (83.27%)			
pf2075	Friendly	4,821	1,751 (36.32%)	1,794 (37.21%)	1,137 (23.58%)	139 (2.88%)			
pf2076	Respectful of one another	5,203	2,207 (42.42%)	1,629 (31.31%)	1,133 (21.78%)	234 (4.50%)			
pf2077	Remote or distant from one another	5,109	110 (2.15%)	425 (8.32%)	1,739 (34.04%)	2,835 (55.49%)			

* For question pf2065, no was coded as missing and therefore we do not have a figure for the number of people

### Childhood happiness and unhappiness

Each parent was asked to rate their memories of happiness/unhappiness at each of three ages: under 6, 6-11 and 12-15 years. As a validation exercise, the mother was asked to do this on two separate occasions, and the data are found in the C files and the D files. Table 6a shows these data, and Table 6b lists the partners’ memories of happiness in their own childhoods as self-reported on one occasion during pregnancy.

**Table 6a.** Mother’s memories of happiness and unhappiness in childhood:

Variable Number	Question	N	Very happy	Moderately happy	Not really happy	Quite unhappy	Very unhappy	Can’t remember
	*Looking back would you call* *your childhood happy?*							
c441	0–5 years	12,305	7,984 (64.88%)	2,068 (16.81%)	175 (1.42%)	47 (0.38%)	55 (0.45%)	1,976 (16.06%)
c442	6–11 years	12,291	7,355 (59.84%)	3,686 (29.99%)	619 (5.04%)	204 (1.66%)	194 (1.58%)	233 (1.89%)
c443	12–15 years	12,293	5,231 (42.55%)	4,796 (39.01%)	1,251 (10.18%)	474 (3.86%)	484 (3.94%)	57 (0.46%)
d760	0–5 years	12,488	8,849 (71.09%)	1,605 (12.89%)	206 (1.65%)	46 (0.37%)	53 (0.43%)	1,689 * (13.57%)
d761	6–11 years	12,448	7,886 (63.35%)	3,224 (25.90%)	668 (5.37%)	194 (1.56%)	185 (1.49%)	291 * (2.34%)
d762	12–15 years	12,448	5,436 (43.67%)	4,529 (36.38%)	1,329 (10.68%)	467 (3.75%)	508 (4.08%)	179 * (1.44%)

* *For the C questionnaire, ‘Can’t Remember’ was coded as a separate category to those who had responded to the questionnaire but had not answered this particular question. Those who had not answered the question were coded as missing data. For the D questionnaire, instances of ‘Can’t Remember’ have been coded as missing data alongside those who did not answer the question. Therefore, for the D questionnaire, we cannot be certain that every response for ‘Can’t Remember’ is a genuine response and not a missing case.*

**Table 6b.** Mother’s partner’s memories of happiness and unhappiness in childhood.

Variable Number	Question	N	Very Happy	Moderately Happy	Not Really Happy	Quite happy	Very Unhappy
	*Looking back would you* *call your childhood happy?*						
pa760	0–5 years	7,183	5,634 (78.44%)	1,381 (19.23%)	111 (1.55%)	20 (0.28%)	37 (0.52%)
pa761	6–11 years	8,218	5,148 (62.64%)	2,515 (30.60%)	367 (4.47%)	101 (1.23%)	87 (1.06%)
pa762	12–15 years	8,309	4,061 (48.87%)	3,119 (37.54%)	724 (8.71%)	237 (2.85%)	168 (2.02%)
pa763*	Overall childhood happiness	8,329	3,685 (44.24%)	3,272 (39.28%)	881 (10.58%)	271 (3.25%)	220 (2.64%)

*The pa763 Overall childhood happiness score is a derived variable computed by adding together the three variables (pa760–pa762) in section F Question 18 of the PA Partners Questionnaire. These questions asked the partner to rate their childhood happiness at 0–5 years (pa760), 6–11 years (pa761) and 12–15 years (pa762)*

### Maternal care score

The Parental Bonding Instrument ^28^ is a scale that seeks to define the principal dimensions of child/parent bonding and to examine the importance of these factors in determining the strength of a child’s relationship with their parent. In 1987, Gamsa ^29^ re-wrote several of the questions in the Parental Bonding Instrument as in the original test, five of the questions contained double negatives which were considered to be confusing to participants.

The ALSPAC team also changed the responses for the questions as piloting revealed that the participants did not like the original options given in the test. Participants were asked to look at statements and rate how like their relationship with their mother the statements were. The answers in the original scale are “Very like”, “Moderately like”, “Moderately unlike” and “Very unlike”. These answers were changed to “Never”, “Sometimes” and “Usually”. In addition, three questions were removed from the test due to their similarity and the perceived repetition causing annoyance to the participants. The responses to each question used are shown in Table 7a and Table 7b for each parent.

**Table 7a.** Variables that make up the Gamsa ^29^ (1987) adapted version of the Parental Bonding Instrument (Parker, Tupling and Brown (1979) ^28^). All variables relate to the period up until the mother was 16 years of age.

Variable Number	Question	N	Never	Sometimes	Usually	Don’t Know	Yes	No
d700	*My mother spoke to me with a warm and* *friendly voice*	12,173	202 (1.66%)	2,368 (19.45%)	9,603 (78.89%)			
d701	*My mother helped me as much as I needed*	12,210	325 (2.66%)	2,087 (17.09%)	9,798 (80.25%)			
d702	*My mother let me do those things I liked* *doing*	12,236	228 (1.86%)	4,930 (40.29%)	7,078 (57.85%)			
d703	*My mother seemed emotionally cold to me*	12,185	9,294 (76.27%)	2,296 (18,84%)	595 (4.88%)			
d704	*My mother appeared to understand my* *problems and worries*	12,211	978 (8.00%)	5,074 (41.53%)	6,165 (50.46%)	<5 (0.01%)		
d705	*My mother was affectionate to me*	12,212	558 (4.57%)	3,097 (25.36%)	8,557 (70.07%)			
d706	*My mother tried to control what I did*	12,205	1,614 (13.22%)	7,494 (61.40%)	3,097 (25.37%)			
d707	*My mother invaded my privacy*	12,189	6,418 (52.65%)	4,780 (39.22%)	991 (8.13%)			
d708	*My mother let me decide things for myself*	12,217	563 (4.61%)	6,122 (50.11%)	5,532 (45.28%)			
d709	*My mother made me feel I wasn’t wanted*	12,219	10,200 (83.48%)	1,541 (12.61%)	478 (3.91%)			
d710	*My mother talked things over with me*	12,223	1,216 (9.95%)	5,584 (45.68%)	5,423 (44.37%)			
d711	*My mother gave me the freedom I wanted*	12,213	899 (7.36%)	6,940 (56.82%)	4,373 (35.81%)	<5 (0.01%)		
d712	*My mother praised me*	12,792	832 (6.82%)	5,012 (41.11%)	6,348 (52.07%)			
							**Yes**	**No**
d713	*My mother enjoyed talking things over with* *me*	11,998					9,648 (80.41%)	2,350 (19.59%)
d714	*My mother frequently smiled at me*	12,104					10,828 (89.46%)	1,276 (10.54%)
d715	*My mother tended to baby me*	12,141					2,360 (19.44%)	9,781 (80.56%)
d716	*My mother seemed to understand what I* *needed or wanted*	11,981					9,323 (77.81%)	2,658 (22.19%)
d717	*My mother could make me feel better when I* *was upset*	12,044					10,390 (86.27%)	1,654 (13.73%)
d718	*My mother felt I could not look after myself* *unless she was around.*	12,097					1,890 (15.62%)	10,207 84.38%)
d719	*My mother let me go out as often as I* *wanted*	12,004					5,484 (45.68%)	6,520 (54.32%)
d720	*My mother was overprotective of me*	12,062					2,537 (21.03%)	9,525 (78.97%)
d721	*My mother let me dress in any way I pleased*	12,041					6,935 (57.59%)	5,106 (42.41%)

**Table 7b.** Variables that make up the Gamsa ^29^ (1987) adapted version of the Parental Bonding Instrument (Parker, Tupling and Brown (1979) ^28^). All variables relate to the period up until the mother’s partner was 16 years of age.

Variable Number	Question	N	Never	Sometimes	Usually	Don’t Know	Yes	No
pa700	*My mother spoke to me with a warm and* *friendly voice*	7,057	105 (1.49%)	1,387 (19.65%)	5,565 (78.86%)			
pa701	*My mother helped me as much as I needed*	7,075	149 (2.11%)	1,208 (17.07%)	5,718 (80.82%)			
pa702	*My mother let me do those things I liked doing*	7,112	68 (0.96%)	2,879 (40.48%)	4,165 (58.56%)			
pa703	*My mother seemed emotionally cold to me*	7,086	5,777 (81.53%)	1,049 (14.80%)	260 (3.67%)			
pa704	*My mother appeared to understand my* *problems and worries*	7,092	451 (6.36%)	2,994 (42.22%)	3,647 (51.42%)			
pa705	*My mother was affectionate to me*	7,104	226 (3.18%)	1,828 (25.73%)	5,050 (71.09%)			
pa706	*My mother tried to control what I did*	7,096	847 (11.94%)	4,219 (59.46%)	2,030 (28.61%)			
pa707	*My mother invaded my privacy*	7,095	3,825 (53.91%)	2,798 (39.44%)	472 (6.65%)			
pa708	*My mother let me decide things for myself*	7,098	223 (3.14%)	3,395 (47.83%)	3,480 (49.03%)			
pa709	*My mother made me feel I wasn’t wanted*	7,098	6,240 (87.91%)	654 (9.21%)	204 (2.87%)			
pa710	*My mother talked things over with me*	7,091	774 (10.92%)	3,909 (55.13%)	2,408 (33.96%)			
pa711	*My mother gave me the freedom I wanted*	7,091	265 (3.74%)	3,484 (49.13%)	3,342 (47.13%)			
pa712	*My mother praised me*	7,090	368 (5.19%)	3,325 (46.90%)	3,397 (47.91%)			
							**Yes**	**No**
pa713	*My mother enjoyed talking things over with me*	6,984					5,483 (78.51%)	1,501 (21.49%)
pa714	*My mother frequently smiled at me*	7,057					6,411 (90.85%)	646 (9.15%)
pa715	*My mother tended to baby me*	7,036					2,056 (29.22%)	4,980 (70.78%)
pa716	*My mother seemed to understand what I needed or wanted*	7,013					5,682 (81.02%)	1,331 (18.98%)
pa717	*My mother could make me feel better when I was upset*	7,022					6,143 (87.48%)	879 (12.52%)
pa718	*My mother felt I could not look after myself unless she was around.*	7,039					1,315 (18.68%)	5,724 (81.32%)
pa719	*My mother let me go out as often as I wanted*	7,027					4,093 (58.25%)	2,934 (41.75%)
pa720	*My mother was overprotective of me*	7,032					1,463 (20.80%)	5,569 (79.20%)
pa721	*My mother let me dress in any way I pleased*	7,021					4,198 (59.79%)	2,823 (40.21%)

From the questions two separate scales were derived as specified by Gamsa indicating (a) maternal care and (b) maternal over-protection. These can be found in Table 8. To create these variables, all coding was reversed for variables d702, d703, d708, d709 and d711 to make the negative response have the higher value. The binary variables d713, d714, d715, d716, d717, d718, d719, d720 and d721 were also recoded so that they faced the same direction.

**Table 8.** Derived continuous variables based on questions asked of each parent about their relationship with their mother during childhood.

Variable Number	Question	N	Range
	**Study mother**		
d724	*Maternal care score using the Parental Bonding Instrument*	11,431	0 – 24
d725	Maternal care score: Modes/no. missing cases *	12,447	0 – 24
d726	*Maternal care score: No. missing items*	12,448	0 – 12
d727	*Maternal over protection score using the Parental Bonding Instrument*	11,545	0 – 20
d728	Maternal over protection score: Modes/no. missing cases *	12,447	0 – 20
d729	*Maternal over protection score: No. missing items*	12,447	0 – 10
	**Study father**		
pa724	*Maternal care score*	6,707	0–24
pa725	Maternal care score – Modes/missing cases *	7,117	0–24
pa726	*Maternal care score –No. missing items*	7,305	0–12
pa727	*Maternal over protection score*	6,819	0–20
pa728	Maternal over protection score – Modes/ missing cases *	7,108	0–20
pa729	*Maternal over protection score – No. missing cases*	7,305	0–10

* *Missing cases were put to the modes of the distribution*

**Figure 3.**
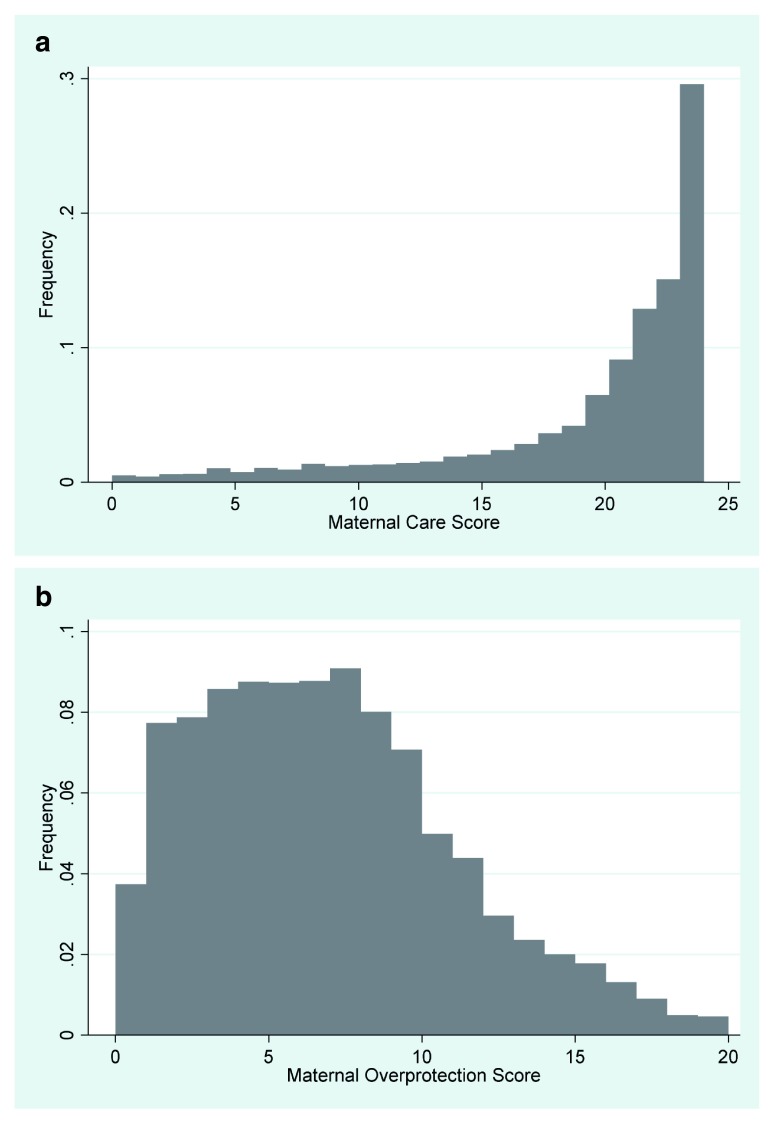
**a.** Maternal Care Score. **b.** Maternal Overprotection Score.

The Maternal Care Score, d724 ( Figure 3a) was created by adding together the responses for variables d700, d701, d703, d704, d705, d709, d710, d712, d713, d714, d716 and d717, then minus 12 from the total. The over protection scale d727 ( Figure 3b) was created by adding together the responses for variables d702, d706, d707, d708, d707, d711, d717, d718, d719, d720 and d721, and then minus 10 from the total. Similar coding and creating of derived variables were undertaken for the paternal scales.

The Maternal Care Score variable comprises a continuous measure of how much the study parent thought their own mother cared for them on a scale of 0–24, with 24 being the most caring mothers. The Overprotection Scale measured how overprotective the study parent thought their own mother was on a scale of 0–20, with 20 being the most overprotective mothers. The numbers of cases in each scale are shown firstly for the scales based on complete data, secondly for scales where the missing response to the individual data have been replaced by the mode for the item, and thirdly the number of such missing items are given.

### Strengths and limitations of the data

The strengths of these data include the large sample size, with over 20,000 participants with data available ^23^. The only inclusion requirements at enrolment for this study were the geographical location the participating mother resided in and the expected date of delivery. The participants recruited to the study were broadly representative of the general population of new parents' resident in the area at the time in terms of sex, ethnicity and socio-economic status ^24^.

All parent participants received the same questions and one of the major strengths of this study is the vast array of other data that is available with several decades of follow up and the opportunity to examine effects across generations. Available data includes information about the study parents’ relationships with the study child, biological markers from parents and children, data regarding the grandparent’s health, life experiences and demographics and data gathered from the grandchildren. This makes the data very flexible and relatable to intergenerational aspects of the family’s life.

A key limitation of the study is the lack of ethnic diversity. At the time of enrolment, the county of Avon was mainly Caucasian, therefore there were too few Black, Asian and Minority Ethnic (BAME) participants (<6% in total) to allow for detailed analysis by ethnic background.

## Data availability

### Underlying data

ALSPAC data access is through a system of managed open access. The steps below highlight how to apply for access to the data included in this paper and all other ALSPAC data.

1. Please read the ALSPAC access policy ( http://www.bristol.ac.uk/media-library/sites/alspac/documents/researchers/data-access/ALSPAC_Access_Policy.pdf) which describes the process of accessing the data and biological samples in detail, and outlines the costs associated with doing so.
2. You may also find it useful to browse our fully searchable research proposals database ( https://proposals.epi.bristol.ac.uk/), which lists all research projects that have been approved since April 2011.
3. Please submit your research proposal ( https://proposals.epi.bristol.ac.uk/) for consideration by the ALSPAC Executive Committee using the online process. You will receive a response within 10 working days to advise you whether your proposal has been approved.
  If you have any questions about accessing data, please email: alspac-data@bristol.ac.uk (data) or bbl-info@bristol.ac.uk (samples).

## Ethical approval and consent

Prior to commencement of the study, approval was sought from the ALSPAC Ethics and Law Committee and the Local Research Ethics Committees ^2^. Informed consent for the use of data collected via questionnaires and clinics was obtained from participants following the recommendations of the ALSPAC Ethics and Law Committee at the time. Questionnaires were completed in the participants own home and return of the questionnaires was taken as continued consent for their data to be included in the study. Full details of the approvals obtained are available from the study website ( http://www.bristol.ac.uk/alspac/researchers/research-ethics/). Study members have the right to withdraw their consent for elements of the study or from the study entirely at any time.
