# Supplementary material for: Traumatic childhood events of parents enrolled in the Avon Longitudinal Study of Parents and Children (ALSPAC)
Source: Wellcome Open Res. 2020 Apr 8;5:65. [Version 1] doi: 10.12688/wellcomeopenres.15804.1 (PMC7199499; doi:10.12688/wellcomeopenres.15804.1)
Supplement: Peer review history

# Peer review: Reviewer response for version 1

**DOI:** 10.21956/wellcomeopenres.17331.r38394

**Published:** 2020-05-04

**Authors:** Barbara Maughan

**Affiliations:** 1 Social, Genetic and Developmental Psychiatry Centre, Institute of Psychiatry, Psychology and Neuroscience, King's College London, London, UK

ALSPAC – the Avon Longitudinal Study of Parents and Children – is an extraordinarily rich resource for the study of human development. Between April 1991 and December 1992 more than 14,000 pregnant women living in the Avon area of south west England were recruited into the study, and these women, their partners, and the children they gave birth to have been studied intensively since that time. The study’s reach is not only long, but also broad: it is truly multidisciplinary, and its archives include medical, social, genetic, psychological and environmental data alongside an extensive range of biomarkers. Not surprisingly, researchers worldwide have seized the opportunity to use these rich data to explore the multitude of factors that go to shape development.

 Even for seasoned users of ALSPAC data, the sheer scope of the study can at times prove daunting. Here, papers such as Ellis et al’s are especially welcome. The authors provide a detailed account of the data collected in one specific area: the ALSPAC parents’ reports, collected in the earliest stages of the study, of their own exposure to traumatic events in childhood. There is already evidence (some based on ALSPAC data) that such exposures show links with adverse developmental outcomes in the *next* generation. From a public health perspective it is vital we understand more about such intergenerational influences, how they are mediated, and – most important of all - how they might best be offset. This highly practical guide should do much to encourage other investigators to use ALSPAC data to seek answers to these questions.

 One word of caution is in order. One of the great strengths of the ALSPAC data-set is that it is *prospective *in nature, charting development and exposures as they unfold. But reports of parents’ childhood experiences are inevitably *retrospective*, reflecting parents’ recall of events and experiences many years in the past. Emerging evidence suggests that retrospective and prospective accounts of childhood adversities are not simply interchangeable: they show moderate agreement, but that agreement is by no means complete (see e.g. Baldwin et al, 2019 ^1^; Newbury et al, 2018 ^2^; Reuben et al, 2016 ^3^). Within the same generation, retrospective reports of childhood adversity relate most strongly to self-reported health outcomes, while prospective reports relate more strongly to objectively observed markers of health. It is unclear at this stage how far (if at all) such differences may impact estimates of intergenerational transmission. As investigators take advantage of the roadmap that Ellis et al provide, however, it would be well for them to have the potential for such variations in mind.

Are sufficient details of methods and materials provided to allow replication by others?

Yes

Is the rationale for creating the dataset(s) clearly described?

Yes

Are the datasets clearly presented in a useable and accessible format?

Yes

Are the protocols appropriate and is the work technically sound?

Yes

Reviewer Expertise:

lifecourse epidemiology

I confirm that I have read this submission and believe that I have an appropriate level of expertise to confirm that it is of an acceptable scientific standard.

## References

[1] : Agreement Between Prospective and Retrospective Measures of Childhood Maltreatment: A Systematic Review and Meta-analysis. *JAMA Psychiatry*.2019;76(6) : 10.1001/jamapsychiatry.2019.0097 584-593 10.1001/jamapsychiatry.2019.0097 30892562PMC6551848

[2] : Measuring childhood maltreatment to predict early-adult psychopathology: Comparison of prospective informant-reports and retrospective self-reports. *J Psychiatr Res*.96: 10.1016/j.jpsychires.2017.09.020 57-64 10.1016/j.jpsychires.2017.09.020 28965006PMC5725307

[3] : Lest we forget: comparing retrospective and prospective assessments of adverse childhood experiences in the prediction of adult health. *J Child Psychol Psychiatry*.57(10) : 10.1111/jcpp.12621 1103-12 10.1111/jcpp.12621 27647050PMC5234278

# Peer review: Reviewer response for version 1

**DOI:** 10.21956/wellcomeopenres.17331.r38392

**Published:** 2020-04-21

**Authors:** Richard E. Tremblay, Sylvana M. Côté, Marie-Pier Larose

**Affiliations:** 1 University of Montreal, Montreal, Canada

The paper by Ellis et al. is an important contribution to one of the best intergenerational longitudinal studies of human bio-psycho-social development, the Avon Longitudinal Study of Parents and Children (ALSPAC). The paper describes the exceptionally large amount of information that was collected, at the time of pregnancy, on the parents’ traumatic events during their own childhood. 

  We thought that we were very familiar with the content of the ALSPAC study before we read the Ellis et al. paper. However, it made us realise the importance of the information collected on the prenatal and perinatal development of both parents. The paper will most certainly convince other investigators who study intergenerational effects during human development to use this exceptional data set. 

 Ellis et al. draw attention to ALSPAC’s abundant information concerning both parents’ life events and mental health during their own childhood and during their child’s development. This is important, because ALSPAC provides a unique opportunity to answer several questions concerning two interrelated dimensions of intergenerational impacts on bio-psycho-social development.

 The first, is related to *assortative mating* processes based on shared life experiences, such as traumatic life events and mental health problems (e.g. Nordsletten et al., 2016 ^1^). The second is *intergenerational epigenetic mechanisms*. There is increasing evidence that traumatic events during an individual’s development can have epigenetic consequences on his children’s bio-psycho-social development (e.g. Jawaid et al., 2019 ^2^; Perez et al., 2019 ^3^). Because epigenetic data on mothers and children is available from birth onwards, for a subsample of the ALSPAC’s children (see Cecil et al., 2014 ^4^ and Houtepen 2019 ^5^), investigators can study the mechanisms implicated in the association between both parents’ adverse childhood events and their children’s epigenetic profile development. Considering the richness of the ALSPAC database, numerous other mediators and moderators can be included in these analyses.

Are sufficient details of methods and materials provided to allow replication by others?

Yes

Is the rationale for creating the dataset(s) clearly described?

Yes

Are the datasets clearly presented in a useable and accessible format?

Yes

Are the protocols appropriate and is the work technically sound?

Yes

Reviewer Expertise:

NA

We confirm that we have read this submission and believe that we have an appropriate level of expertise to confirm that it is of an acceptable scientific standard.

## References

[1] : Patterns of Nonrandom Mating Within and Across 11 Major Psychiatric Disorders. *JAMA Psychiatry*.2016;73(4) : 10.1001/jamapsychiatry.2015.3192 354-61 10.1001/jamapsychiatry.2015.3192 26913486PMC5082975

[2] : Inter- and transgenerational inheritance of behavioral phenotypes. *Current Opinion in Behavioral Sciences*.2019;25: 10.1016/j.cobeha.2018.12.004 96-101 10.1016/j.cobeha.2018.12.004

[3] : Intergenerational and transgenerational epigenetic inheritance in animals. *Nat Cell Biol*.21(2) : 10.1038/s41556-018-0242-9 143-151 10.1038/s41556-018-0242-9 30602724

[4] : Environmental risk, Oxytocin Receptor Gene (OXTR) methylation and youth callous-unemotional traits: a 13-year longitudinal study. *Mol Psychiatry*.2014;19(10) : 10.1038/mp.2014.95 1071-7 10.1038/mp.2014.95 25199917PMC4231290

[5] : Childhood adversity and DNA methylation in two population-based cohorts. *Translational Psychiatry*.2018;8(1) : 10.1038/s41398-018-0307-3 10.1038/s41398-018-0307-3 30510187PMC6277431
